# Adaptive Optics Retinal Imaging in *RDH12*-Associated Early Onset Severe Retinal Dystrophy

**DOI:** 10.1167/iovs.65.3.9

**Published:** 2024-03-11

**Authors:** Malena Daich Varela, Mira Dixit, Angelos Kalitzeos, Michel Michaelides

**Affiliations:** 1 Moorfields Eye Hospital, London, United Kingdom; 2 UCL Institute of Ophthalmology, University College London, London, United Kingdom

**Keywords:** adaptive optics, RDH12, retinal imaging, inherited retinal diseases, genetics

## Abstract

**Purpose:**

*RDH12* is among the most common genes found in individuals with early-onset severe retinal (EOSRD). Adaptive optics scanning light ophthalmoscopy (AOSLO) enables resolution of individual rod and cone photoreceptors in the retina. This study presents the first AOSLO imaging of individuals with *RDH12*-associated EOSRD.

**Methods:**

Case series of patients who attended Moorfields Eye Hospital (London, UK). Spectral-domain optical coherence tomography, near-infrared reflectance (NIR), and blue autofluorescence imaging were analyzed. En face image sequences of photoreceptors were recorded using either of two AOSLO modalities. Cross-sectional analysis was undertaken for seven patients and longitudinal analysis for one patient.

**Results:**

Nine eyes from eight patients are presented in this case series. The mean age at the time of the assessment was 11.2 ± 6.5 years of age (range 7–29). A subfoveal continuous ellipsoid zone (EZ) line was present in eight eyes. Posterior pole AOSLO revealed patches of cone mosaics. Average cone densities at regions of interest 0.5° to the fovea ranged from 12,620 to 23,660 cells/mm^2^, whereas intercell spacing ranged from 7.0 to 9.7 µm.

**Conclusions:**

This study demonstrates that AOSLO can provide useful high-quality images in patients with EOSRD, even during childhood, with nystagmus, and early macular atrophy. Cones at the posterior pole can appear as scattered islands or, possibly later in life, as a single subfoveal conglomerate. Detailed image analysis suggests that retinal pigment epithelial stress and dysfunction may be the initial step toward degeneration, with NIR being a useful tool to assess retinal well-being in *RDH12*-associated EOSRD.

Early-onset severe retinal dystrophy (EOSRD) is a rare genetic condition that causes profound visual impairment from early in life.^1^^,^^2^ It affects roughly one in 80,000 children, accounting for around 5% of all inherited retinal diseases (IRD).^3^ Patients can have isolated EOSRD, or it can occur in the context of a syndrome, affecting other body systems (e.g., Joubert, Senior Loken). EOSRD's inheritance pattern is predominantly recessive, and currently more than 25 causative genes have been identified (https://web.sph.uth.edu/RetNet/). The pathophysiology of EOSRD generally entails a protein with a function in retinal development, the visual cycle, or the survival of photoreceptors or retinal pigment epithelium (RPE) cells.^4^ When mutated, this protein either becomes abnormal/toxic, scarce, or absent, resulting in impaired functioning of the outer retina and ultimately leading to cellular death.

Retinal imaging such as optical coherence tomography (OCT) and fundus autofluorescence (FAF) are key to characterizing and monitoring EOSRD.^5^ OCT shows in vivo cross-sectional views of the retinal layers, whereas FAF uncovers the retina's health and metabolism. However, clinical OCT does not enable resolution of individual cells. Adaptive optics scanning light ophthalmoscopy (AOSLO) uses adaptive optics to measure and correct for higher-order aberrations in the eye.^6^^,^^7^ Although AOSLO is not currently incorporated as part of standard clinical practice, this imaging modality is associated with improved lateral resolution, thus enabling the visualization of individual cone and rod photoreceptors in the retina noninvasively and in real time.^8^ Another advantage of AOSLO is the ability to generate confocal and nonconfocal images. Confocal AOSLO image signals are thought to rely upon photoreceptors with intact outer segments that can propagate light. Therefore the identification of photoreceptors using confocal AOSLO may be more challenging for patients with retinal diseases that impact the outer segment. In these cases, the cone mosaic can still be visualized using nonconfocal AOSLO, even in pathological conditions, where the light-propagating photoreceptor outer segments may be compromised.^9^

AOSLO has been beneficial for the study of a wide range of retinal diseases, including age-related macular degeneration,^10^^,^^11^ diabetic retinopathy,^12^ glaucoma,^13^^,^^14^ and IRD.^9^^,^^15^ Important clinical insights include but are not limited to the finding that increased axial length lowers the number of cones per unit of surface,^16^ the detailed subcellular analysis of photoreceptor inner and outer segments integrity,^17^ the clarification of the primary site of degeneration (RPE vs. photoreceptors) in diseases such as choroideremia and Stargardt,^18^^–^^20^ and even the monitoring of changes in the cone mosaic after gene therapy in individuals with IRD.^21^^,^^22^

*RDH12* (MIM* 608830) is a gene involved in clearing toxic compounds such as excess all-trans retinal and its by-products (e.g., A2E and 4-HNE) from the photoreceptor inner segments.^23^ It has a wide phenotypic spectrum, being associated mostly with EOSRD and less frequently with retinitis pigmentosa (RP), cone/cone-rod dystrophy, and macular dystrophy.^24^ *RDH12*-EOSRD usually starts with nystagmus and poor vision before four years of age, and patients often become severely visually impaired after age 10 years.^25^ *RDH12* is among the top five most frequent genes to cause EOSRD, along with *GUCY2D*, *CEP290*, *CRB1*, and *RPE65*.^25^^,^^26^ The large majority of *RDH12* variants are inherited in an autosomal recessive pattern; however, damaging variants altering the reading frame-specific C-terminal peptide have been associated with autosomal dominant RP.^27^^,^^28^ AOSLO imaging has been reported in a 32-year-old patient with *RDH12*-associated ADRP, where split-detection AOSLO suggested the presence of cellular structures in the ellipsoid zone (EZ) island and possibly beyond this area.^27^

This study presents the first AOSLO imaging of individuals with *RDH12*-associated EOSRD. High-quality retinal imaging of individuals with profound vision loss and nystagmus is inherently challenging; however, AOSLO and image post-processing techniques that correct for eye motion enabled us to gain insight into photoreceptor structure in a cohort of patients aged seven to 29 years old. The findings from this study are a valuable addition toward the understanding of this still not fully elucidated gene^23^ and the characterization of this severe retinopathy, with implications for anticipated clinical trials.

## Methods

This is a case series of patients who attended Moorfields Eye Hospital (London, UK). All patients were diagnosed with *RDH12*-associated EOSRD, had biallelic disease-causing variants in *RDH12*, best-corrected visual acuity greater than LogMAR 1.3 in at least one eye, were aged seven years or older, and were able to participate in OCT and AOSLO assessments. Informed consent was obtained from the adult patient and parents, and assent from pediatric patients. Ethical approval was provided by the local ethics committee, and the study honored the tenets of the Declaration of Helsinki. Cross-sectional analysis was undertaken for seven patients and longitudinal analysis for one patient.

GraphPad Prism 8.0.2 (GraphPad Software, San Diego, CA, USA) was used for statistical analysis. The threshold of significance was set at *P* < 0.05. All continuous parameters passed the Kolmogorov-Smirnov or Shapiro-Wilk normality tests (*P* > 0.1), and their associations were assessed with linear regressions.

### OCT and FAF Imaging

All patients had spectral-domain OCT (SD-OCT) and near-infrared reflectance imaging (NIR; Heidelberg Spectralis; Heidelberg Engineering, Inc., Heidelberg, Germany). Four patients also had blue autofluorescence (BAF) with the Heidelberg device, whereas the other four did not tolerate it. All patients underwent color and green autofluorescence ultrawide-field retinal photography (Optos PLC, Dunfermline, UK). Optos images were assessed and analyzed when Heidelberg imaging was not available (n = 4). Fovea-centered horizontal and vertical macular volume scans were performed in a 6 mm^2^ nominal area that included the 1-, 3-, and 6-mm grid templates from the Early Treatment Diabetic Retinopathy Study. Central macular thickness (CMT) corresponds to the central 1mm area. Inner limiting membrane and Bruch's membrane were automatically segmented by the manufacturer software (Heyex version 1.9.14.0; Heidelberg Engineering) and adjusted manually as needed by a trained ophthalmologist (M.D.V.). Ellipsoid zone (EZ) width and outer nuclear layer thickness (ONLT) were measured manually while images were displayed in a 1:1 µm setting at the foveal scan. To correct for ocular magnification, we linearly scaled each transverse measurement using patients’ axial length (IOLMaster 700, Carl Zeiss Meditec, Germany).^29^

### Adaptive Optics Scanning Light Ophthalmoscopy

#### Image Acquisition

En face image sequences of photoreceptors were recorded using either of two AOSLO devices.^30^ Confocal reflectance imaging revealed photoreceptor outer segments using a 790 nm superluminescent diode (Superlum, Cork, Ireland) whereas nonconfocal multiple-scattered light revealed photoreceptor inner segments using either a split-^17^ or a quadrant-detection setup.^31^ The light source for wavefront-sensing was an 850 nm superluminescent diode (Superlum), and the wavefront correction was performed by a Hi-Speed DM97-15 deformable mirror (Alpao, Montbonnot-Saint-Martin, France). Patient's pupils were dilated using a drop of 1% tropicamide and 2.5% phenylephrine each. They were stabilized using a bite-bar while asked to fixate on the center of a cross for about nine seconds at a time for a single retinal location. Overlapping retinal locations were recorded so these could later be montaged either manually or automatically.^32^ Images were obtained using either a 1° or 1.5° field of view.

The AOSLO imaging protocol aimed to capture the area amounting to the foveal avascular zone, containing any remnant cone photoreceptors; however, this was not always possible for this cohort. The location of the foveal reflex (sometimes observed when adjusting AO focus) can be used as a marker for the anatomical fovea; however, this step was more challenging here because of issues with poor fixation and nystagmus. Additionally, because most patients were very young in age, the timeframe for imaging needed to be reduced to reflect aspects such as concentration levels, fatigue, or willingness to continue. AOSLO sessions took place at the end of the research visit, after other imaging and visual function assessments, and lasted between 15 and 45 minutes per eye. Children were encouraged to pick music during the sessions to make the experience more enjoyable.

#### Post-Processing

Similarly to OCT transverse measurements, AOSLO images were also scaled accordingly. Inherent scanning image distortions were removed by recording a grid of horizontal and vertical lines of known spacing. Reference frames from each image sequence were selected either manually or automatically^33^ to register a number of them and, hence, increase the signal-to-noise ratio as previously described.^34^ Intraframe motion correction (de-warping) was then applied to remove any residual distortion.^35^

For quadrant-detection, beyond the “traditional” vertical split-detection (90°), we also calculated the horizontal (180°), 45° and 135° split-detection images. These four split directions were further processed using the emboss (edge-enhancing) filter in Photoshop CS6 (Adobe Systems, San Jose, CA, USA).^31^ The embossed quadrant-detection image was the primary modality used for cell annotations (where available) because it allowed better resolution of both waveguiding and nonwaveguiding cells, compared with confocal or split-detection modalities, respectively.

AOSLO montages were scaled and manually aligned to NIR/OCT images, using Adobe Illustrator (Adobe Systems Inc.) (see Supplementary Figs. S1–S10). Alignment was done by using visual landmarks identified in dark field AOSLO that corresponded with the NIR images. Horizontal and vertical OCT scans taken at the anatomical fovea (corroborated by the hyperreflective dot at the ILM/vitreous interface or by examining adjacent B-scans) were used to generate a crosshair that in turn acted as a marker for the foveal center in the AOSLO images.

#### Cone Photoreceptor Metrics

Cone locations for all datasets were manually annotated by two observers (A.K. and M.D.) using Mosaic Analytics (Translational Imaging Innovations Inc., Hickory, NC, USA). To avoid bias, these were performed independently, and observers were masked from the results throughout the process. Where AOSLO imaging encompassed the fovea (n = 6 datasets), cone density could be assessed using region of interests (ROIs) at the foveal center and 0.5° away from it, in all meridians where data were available (see Supplementary Figs. S1, S3–S5, S7, and S8). In some instances, the ROI location needed to be adjusted by no more than 0.25° to ensure better quality images. Within each 100 µm square ROI, all cells were marked producing a list of coordinates’ pairs. Total bound cone density (i.e., cone locations with Voronoi regions that were fully contained within an ROI; cells/mm^2^) and inter-cell distance (µm) was calculated.^36^ These are reported as the average values from each observer (± SD) (Table 1).

**Table 1.** Average Cone Photoreceptor Density and Intercell Spacing ± SD Calculated by the Annotations of Two Graders (A.K. and M.D.) at the Intersection of the Transfoveal OCT Scans and 0.5° Away in All Meridians (Where Possible)

	Bound Cone Photoreceptor Density (Cells/mm^2^) ± SD					Inter-Cell Spacing (nm) ± SD				
Patient ID	Fovea	0.5° Nasal	0.5° Temporal	0.5° Superior	0.5° Inferior	Fovea	0.5° Nasal	0.5° Temporal	0.5° Superior	0.5° Inferior
MM_0572 OD	13,470 ± 5040	16,680 ± 1730	19,710 ± 720	N/A	12,620 ± 750	9.4 ± 1.3	8.6 ± 0.4	7.9 ± 0.0	N/A	9.7 ± 0.5
MM_0628 OS	18,890 ± 6730	N/A	N/A	N/A	N/A	8.3 ± 1.5	N/A	N/A	N/A	N/A
MM_0608 OD	15,770 ± 4060	13,270 ± 160	N/A	13,170 ± 1650	N/A	8.9 ± 1.2	9.6 ± 0.0	N/A	9.7 ± 0.7	N/A
MM_0594 OD V1	22,400 ± 1,910	16,330 ± 1,100	18,630 ± 1,630	20,110 ± 1,740	21,280 ± 2,230	7.4 ± 0.4	8.3 ± 0.6	8.2 ± 0.3	7.8 ± 0.2	7.5 ± 0.4
MM_0594 OS V1	N/A	N/A	19,870 ± 710	18,460 ± 1,330	19,370 ± 4,120	N/A	N/A	7.7 ± 0.0	7.9 ± 0.3	7.9 ± 0.8
MM_0594 OD V2	18,360 ± 1,350	12,870 ± 920	19,440 ± 260	19,090 ± 1,590	23,660 ± 5,440	8.1 ± 0.2	9.5 ± 0.2	8.0 ± 0.1	8.0 ± 0.4	7.0 ± 0.2

Regions of interest were 100 µm square. All annotations were performed on quadrant-detection images apart from MM_0572 where only split-detection was available. For context, estimates of cone density and intercell distance previously reported in unaffected eyes at 150 µm eccentricity (roughly 0.5° from fovea) were 87,000 cells/mm^2^ and 3 µm, respectively; these values reflect the average of 20 subjects with normal trichromatic vision, median age: 23.5 years, range 9–67 years (Cooper RF, Wilk MA, Tarima S, Carroll J. Evaluating Descriptive Metrics of the Human Cone Mosaic. *Invest Ophthalmol Vis Sci*. 2016;57:2992-3001).

#### Longitudinal Analysis

One patient (MM_0594) had available data in the same eye (OD) at two timepoints that were acquired six months apart. The two AOSLO montages were manually scaled and overlayed. Because no consistent anatomical landmarks could be identified in the confocal and quadrant-detection image modalities, dark-field AOSLO was used to guide the overlay. A large ROI was selected encompassing the foveal center with its center shifted 213 µm nasally.

## Results

### Demographics

Nine eyes from eight patients are presented in this case series. Seven patients were children, and one was an adult. The mean age at the time of the assessment was 11.2 ± 6.5 years of age (range 7–29, median 9, Table 2). Four patients were male and four, female.

**Table 2.** Age, Axial Length, OCT Measurements, and Genetics in the Cohort

ID	Age (y)	Eye	Axial Length (mm)	EZA (mm^2^)	Subfoveal EZW (µm)	ONLT (µm)	CMT (µm)	Preserved AF Area (mm^2^)	IR Island (mm^2^)	Genetics
MM_0600	10	OD	21.27	NA	128	0	247	Hypo^†^	Unclear	c.464C>T, p.Thr155Ile (Hom)
MM_0572	13	OD	22.07	0.28	454	80	231	0.38	0.34	c.806_810delCCCTG p.(Ala269GlyfsTer2) & c.680_684delinsT p.(Ala227ValfsTer50)
MM_0576	8	OD	20.74	0.06	171	49	245	Hypo^†^	0.03	c.325G>C, p.Ala109Pro & c.677A>G, p.Tyr226Cys
MM_0628	29	OS	21.34	0.15	317	19	213	0.1	0.21	c.316C>T, p.Arg106Ter & c.142A>G, p.Asn48Asp
MM_0608	9	OD	21.36	0.36	Discont.	49	206	1.57	1.38	c.883C>T, p.Arg295Ter & c.806_810delCCCTG, p.Ala269GlyfsTer2
MM_0594	8	OD	21.24	0.12	396	33	170	0.27	1.96	c.601T>C, p.Cys201Arg (Hom)
MM_0594^*^	8	OD	21.27	0.11	390	29	167	0.25	1.96	
MM_0594	8	OS	21.21	0.13	310	34	173	2.03	1.9	
MM_0629	7	OS	22.07	NA	302	48	198	0.8^†^	0.47	c.632C>T, p.Thr211Ile (Hom)
MM_0642	9	OS	19.07	0.04	54	0	225	Hypo^†^	0.07	c.806_810delCCCTG, p.Ala269GlyfsTer2 (Hom)

AF, autofluorescence; Discont, discontinuous; EZA, ellipsoid zone area; EZW, ellipsoid zone width; Hom, homozygous; IR, infrared; ONLT, outer nuclear layer thickness.

EZ width values have been linearly scaled to correct retinal magnification as follows: measured EZW * (axial length/24.385).^29^

* Measurements at six-month follow-up.

† Performed with Optos. Subfoveal EZW was calculated with horizontal scans.

### OCT, NIR, and BAF Analysis

**Figure 1.**
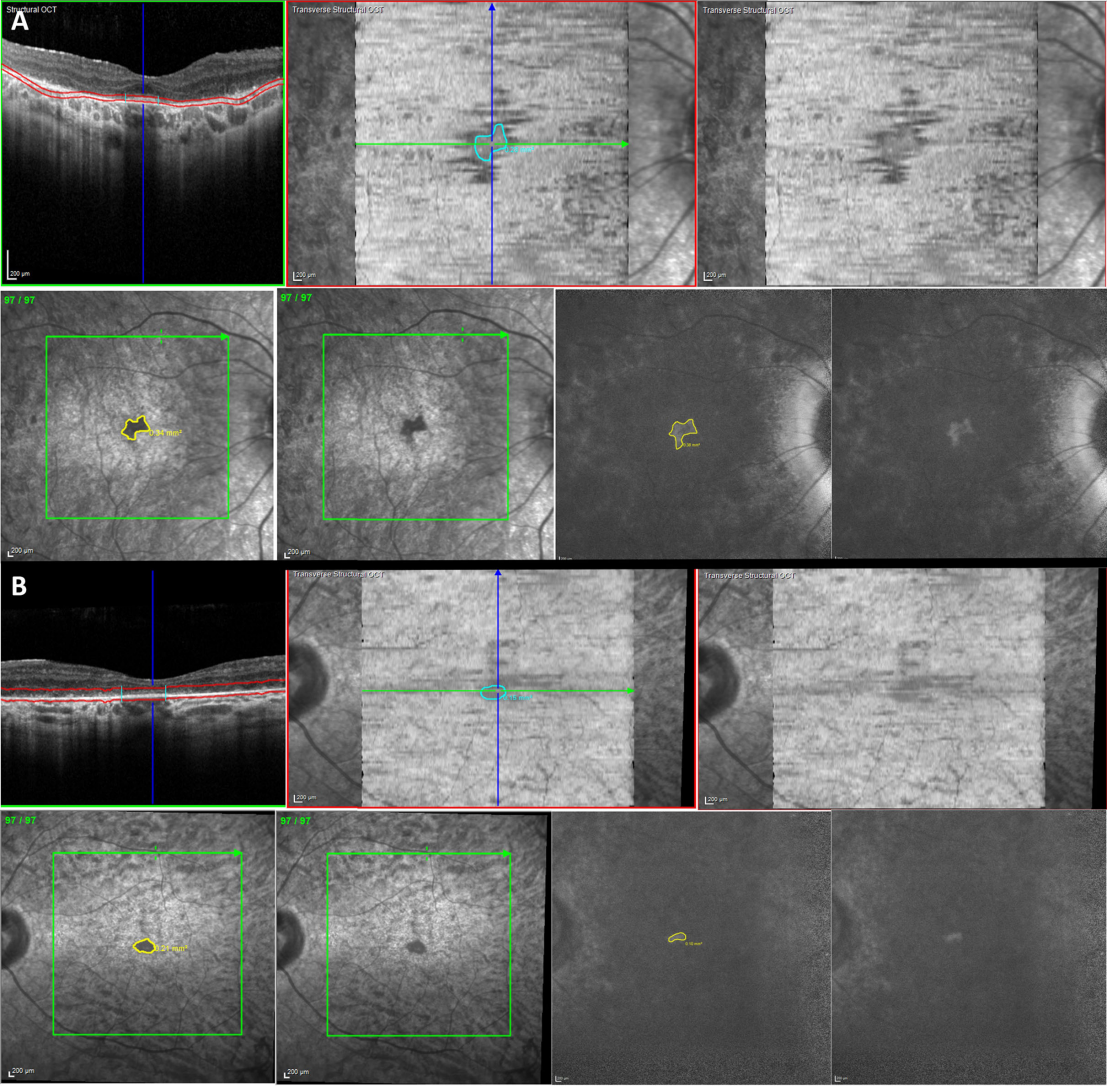
Macular OCT, NIR, and BAF imaging of patients with *RDH12*-EOSRD. (**A**) Right eye of 13-year-old boy (MM_0572), where there is a continuous subfoveal EZ line and a demarcated EZ area by OCT analysis. In NIR, the EZ area matches with a central hyporeflective region, and a sector of spared autofluorescence in BAF. (**B**) Left eye of 29-year-old man (MM_0628), where there is a small subfoveal EZ line and area, with a matching hyporeflective sector in NIR and preserved autofluorescence in BAF.

All patients had peripapillary sparing on FAF. An EZ area of 0.16 ± 0.1 mm^2^ (mean ± SD, median 0.14 mm^2^, Fig. 1) was discernible in seven eyes of six patients. Subfoveal continuous EZ line was present in eight eyes and discontinuous in one; the mean width was 303.2 ± 141.2 µm (median 345 µm, Table 2). CMT was 212 ± 26.6 µm (median 212 µm), and ONLT was 34.6 ± 24.2 µm (median 34.7 µm).

Eight eyes of seven patients had a circumscribed area of decreased NIR in the posterior pole of 0.8 ± 0.8 mm^2^ (mean ± SD, range 0.03–1.96, median 0.47). In seven eyes, this area coincided with the region of continuous and preserved EZ. This area appeared with normal BAF in six eyes (sometimes as pseudo-hyperautofluorescent because of the contrast with the surrounding hypoautofluorescence), and as hypoautofluorescent in the remaining two eyes (Fig. 1). Longitudinal analysis of MM_0594 at a six-month follow-up visit showed no significant differences with baseline assessments (Table 2).

### Photoreceptors Phenotyping

For one patient (MM_0642) transfoveal OCT scans were of low image quality because of nystagmus and from a different time point (two months later); hence, a precise AOSLO foveal center could not be determined (Supplementary Fig. S10); this patient was not included in cone metrics estimations. Cone density estimates for patients with available data are reported in Table 1. Average (± SD) cone densities at ROIs 0.5° to the fovea, ranged from 12,620 (±750) to 23,660 (±5440) cells/mm^2^, whereas intercell spacing ranged from 7.0 (±0.2) to 9.7 (±0.7) µm. Densities were highly variable between datasets and, in some cases, also varied across the respective meridians for an individual. Comparatively, average cone density and intercell distance previously reported in an unaffected eye at 150 µm eccentricity (roughly 0.5° from fovea) were 87,000 cells/mm^2^ and 3 µm, respectively.^36^ Overall, the densities in this *RDH12*-EOSRD population were considerably lower than those reported in similar locations in an unaffected eye but do illustrate varying levels of preserved structure, regardless of age. Notably, between patches of cone mosaics, there were large-in-diameter, contoured bumps that were observed both near the fovea and at more eccentric locations (see examples of this in Supplementary Figs. S3, S6, and S8). Additionally, in some cases, there were cones in the quadrant-detection image that seemed to have a stretched appearance, and this was most apparent in images for MM_0642 (see Supplementary Fig. S10). It is possible that the appearance of stretched photoreceptors could have been due to an imaging artefact, potentially caused by depth of focus or large changes in thickness of inner retinal layers between imaging locations.

**Figure 2.**
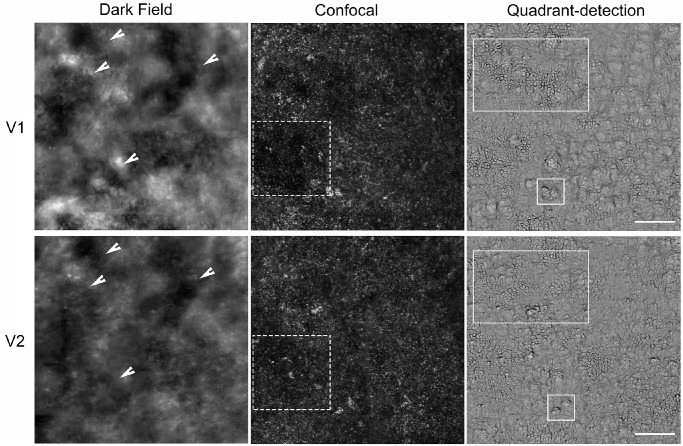
AOSLO imaging of the right eye of patient MM_0594 with *RDH12*-EORSD. Dark-field, confocal, and quadrant-detection images acquired six months apart at visit 1 (V1) and visit 2 (V2) are shown. In the first column, landmarks identified in dark-field imaging are highlighted using *white arrows*—these were used to overlay the images and to allow maximal overlap of the ROI sampling area at the two timepoints. In the second column, dark spaces in confocal images appear to change in location and size, possibly reflecting movement of photoreceptor cells or a reduction in cells resulting in more spacing (*white dashed squares*). In the third column, clusters of cells and individual contoured bumps in quadrant-detection images appear to move location over time (*white solid rectangles*). *Scale bar*: 100 µm.

For the longitudinal data (MM_0594), cone densities assessed using 100 µm square ROIs appeared similar at baseline and six months (Table 1). In nasal and temporal meridians, the count was numerically lower by six months, whereas the opposite was the case in the other meridians. Small variations in counts in either direction are to be expected, because the OCT-derived marker for the foveal center (and therefore the corresponding ROIs) may have fallen at slightly different locations. Additionally, possible changes in cell location and size make it more challenging to track individual cones over time, so comparisons of cone densities here may not be entirely reliable. In Figure 2, images taken at visits six months apart were overlayed, and a larger ROI was used, allowing for a greater overlap of the sampling area at the two timepoints. These images more clearly demonstrate the potential structural changes over the short observational timeframe. For example, clusters of remnant cells appear to move location or alternatively reduce in area and become more spaced out over time.

### Associations Between Cone Density, Age, and OCT/NIR/BAF Findings

There was no significant association between cone photoreceptor density and intercell spacing and any OCT parameter (*P* = 0.06–0.5). Age was also not significantly associated with AOSLO (*P* = 0.9) or OCT parameters (*P* = 0.3–0.8). Regarding OCT parameters, EZ width was significantly associated with EZ area (*P* = 0.03), and CMT with IR island area (*P* = 0.0007).

## Discussion

In this article, we present AOSLO imaging of children and one adult with *RDH12*-EOSRD for the first time, providing insights into the phenotype and natural history of this condition. An organized, rather preserved overall cone mosaic was reported in three adults (Kalitzeos, *IOVS*, 2019;60:ARVO E-Abstract 4584) and one adolescent with *RPE65*-EOSRD.^21^ No further reports exist of AOSLO in individuals with EOSRD to date. It is expected that the features are similar to those found in RP but more aggressive and earlier in life.

### Clinical Relevance of NIR Imaging in *RDH12*-EOSRD

Retinal histologic study of *Rdh12*^−/−^ animal models has shown shortened photoreceptors that have a reduced capacity for retinoid processing,^37^^,^^38^ and enlarged phagosomes in the RPE.^39^ The latter has been associated with suboptimal RPE phagocytosis of photoreceptor outer segments, causing toxicity and subsequent cell death.^40^^,^^41^

Melanin inside RPE cells functions as an oxidative stress scavenger, contributing to retinal well-being.^42^ It has been described that when distressed, RPE cells may initially lose melanin activity, later progress to reduced phagocytotic activity, and lastly die.^43^ Melanin is mostly captured by 787 nm emissions, used by NIR imaging.^44^ NIR imaging has been shown to be more sensitive than BAF at detecting geographic atrophy and pigment migration, whereas BAF is better at characterizing areas with photoreceptor loss and intact RPE.^45^ The fact that patients in this case series had preserved EZ and RPE per OCT but decreased NIR reflectance may indicate that RPE toxicity is possibly the first (or an early) step in the chain of events toward retinal degeneration. This is also supported by the corresponding area of preserved BAF, in keeping with surviving photoreceptors. This would represent another example of a photoreceptor enzyme causing dysregulation and death of RPE cells, like *ABCA4*.^46^ It is interesting that CMT was significantly associated with IR island area only and possibly highlights the use of NIR as an important imaging technique for this disorder.

### Clinical Relevance of AOSLO Imaging in *RDH12*-EOSRD

In our study population, AOSLO imaging in patients with *RDH12*-associated EOSRD demonstrated severe loss in the cone mosaic, with the remaining cells appearing both in scattered islands throughout the posterior pole (MM_0594) or as a single subfoveal conglomerate (MM_0572 and MM_0628, the two oldest patients of the cohort). The presentation was severe, with disorganized and compromised photoreceptors throughout the posterior pole, both in areas with EZ and FAF present and absent. These results contrast with AOSLO imaging findings from a prior study of a patient with autosomal dominant *RDH12*-associated RP, who showed relatively normal foveal cone morphology and density across the EZ island and possibly beyond.^27^

**Figure 3.**
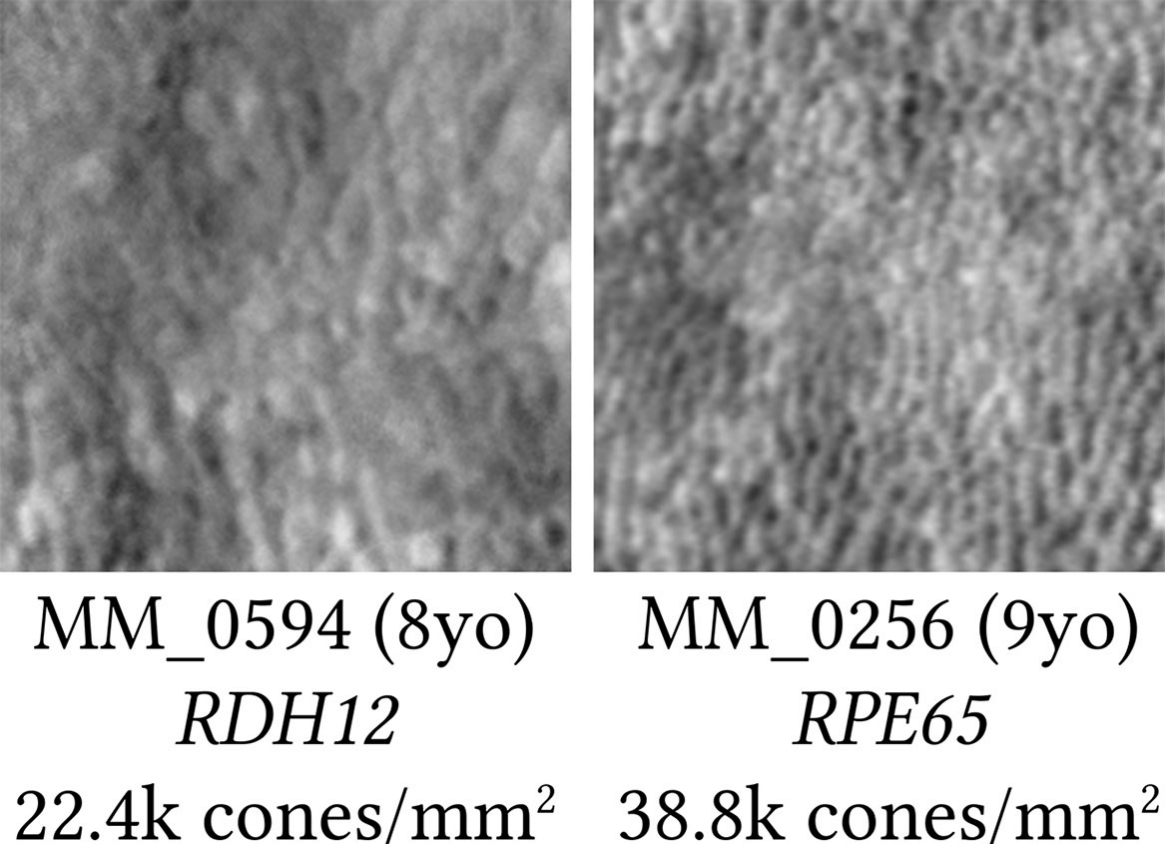
Representative *RDH12*- and *RPE65*-EOSRD cone photoreceptor inner segments phenotype comparison. Both images are 100 µm square taken at the foveal center.

Figure 3 provides a phenotypic comparison of AOSLO images for a representative case of a child with *RPE65*-EOSRD versus *RDH12*-retinal dystrophy; here the child with *RPE65*-retinal dystrophy appears to have largely preserved structure, this being one of the reasons why this gene became a successful target for gene supplementation therapy.^47^ *RDH12* corresponds to the group of EOSRD genes that cause early severe macular and generalized retinal involvement,^23^ so notably the presence of macular photoreceptors captured by AOSLO is a promising finding that points to viable macular cells that could respond to possible gene supplementation therapy in young patients/children.^48^

Retinal imaging in children can be challenging because of limited attention span, difficulty with sustained fixation, and shorter axial length.^40^ Patients with nystagmus or unstable fixation may pose additional challenge. Although AOSLO has been investigated in healthy children in prior studies,^41^ there has been a distinct lack of reports on the use of this advanced imaging technique in young patients with nystagmus until now. The ability to use AOSLO to observe microscopic retinal changes in the presence of pathology noninvasively and in real time can be hugely beneficial to gain insight into therapeutic efficacy and safety, as well as in the identification of patients who may be most likely to benefit from novel treatments. This case series supports the feasibility of AOSLO to observe cone photoreceptors and structural changes over time in patients with EOSRD who can participate in this assessment. EOSRD is at the forefront of gene therapy and given the current demand for novel endpoints to demonstrate the efficacy of potential treatments, AOSLO may become a valuable tool that could potentially enhance the current landscape.

Despite the general success of AOSLO imaging to resolve photoreceptors in young and adult patients with nystagmus, the severity of the condition manifests in heavily disrupted outer and inner segments that makes robust identification of individual cells, challenging—especially across different timepoints. The recent introduction of quadrant detection is a step toward more reliable cell annotation. However, even experienced graders have no prior experience in either this phenotype or in this nonconfocal modality. For this reason, the cone densities reported herein should be considered as estimates. Furthermore, it should be noted that structural integrity of cone inner segments does not prove functional integrity, and the inability to potentially resolve some photoreceptors does not mean that these cells do not exist. In the future, pupil tracking could mitigate the considerable involuntary eye movements in individuals with *RDH12*-EOSRD, thus increasing the number of patients we can gain an insight from in this rare condition.

## Conclusions

This study demonstrates that AOSLO can provide useful high-quality images in patients with EOSRD, even during childhood, with nystagmus and early macular atrophy. Cones at the posterior pole may appear as scattered islands or, later in life, as a single subfoveal conglomerate. Detailed image analysis suggests that RPE stress and misfunction could be the initial step toward degeneration, with NIR becoming a useful tool to assess retinal well-being in *RDH12*-associated EOSRD.

## Supplementary Material

Supplement 1
