# Supplementary material for: Adaptive Optics Retinal Imaging in RDH12-Associated Early Onset Severe Retinal Dystrophy
Source: Invest Ophthalmol Vis Sci. 2024 Mar 11;65(3):9. doi: 10.1167/iovs.65.3.9 (PMC10929749; doi:10.1167/iovs.65.3.9)
Supplement: Supplement 1 [file iovs-65-3-9_s001.pdf]

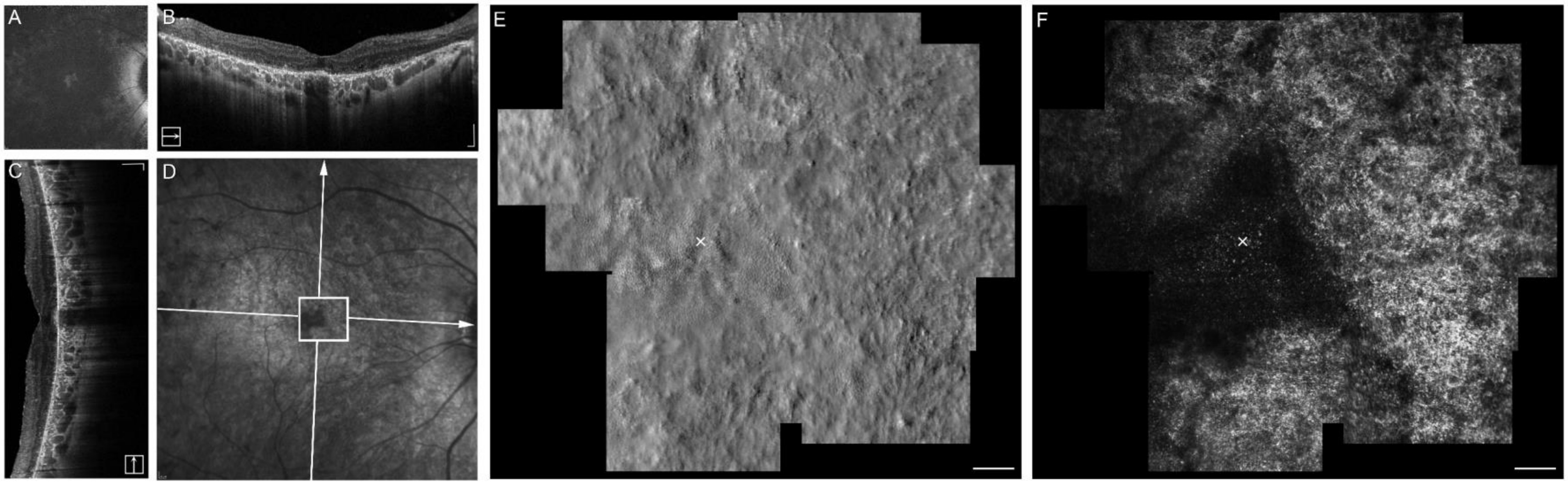

Supplementary Figure 1: Multimodal imaging of the right eye of patient MM_0572 with *RDH12*-EORSD. BAF image shown in (**A**). The horizontal and vertical line scans on the NIR reflectance image in (**D**) indicate the locations of horizontal and vertical OCT B-scans (**B** and **C**, respectively), while the white rectangle represents the area where the AOSLO split-detection image (**E**) and confocal image (**F**) were taken. The intersection of the two line scans marks the fovea; X in images (**E**) and (**F**). Scale bars for (**B**), (**C**) and (**D**) = 200 μm. Scale bars for (**E**) and (**F**) = 100 μm.

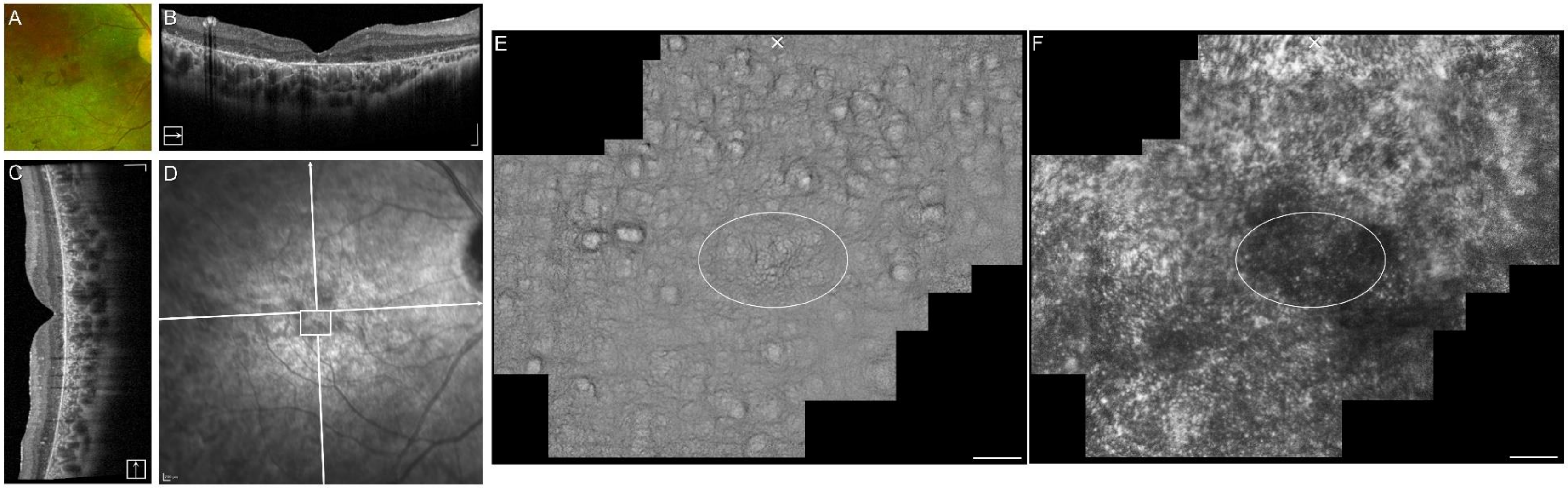

Supplementary Figure 2: Multimodal imaging of the right eye of patient MM_0576 with *RDH12*-EORSD. OPTOS image shown in (**A**) is cropped to reflect the area of the NIR reflectance image in (**D**). The horizontal and vertical line scans in (**D**) reflect the locations of horizontal and vertical OCT B-scans (**B** and **C**, respectively), while the white rectangle represents the area where the AOSLO quadrant-detection image (**E**) and confocal image (**F**) were taken. The white oval highlights the only area identified with remnant photoreceptors. The intersection of the two line scans marks the fovea; X in images (**E**) and (**F**). Scale bars for (**B**), (**C**) and (**D**) = 200 μm. Scale bars for (**E**) and (**F**) = 100 μm.

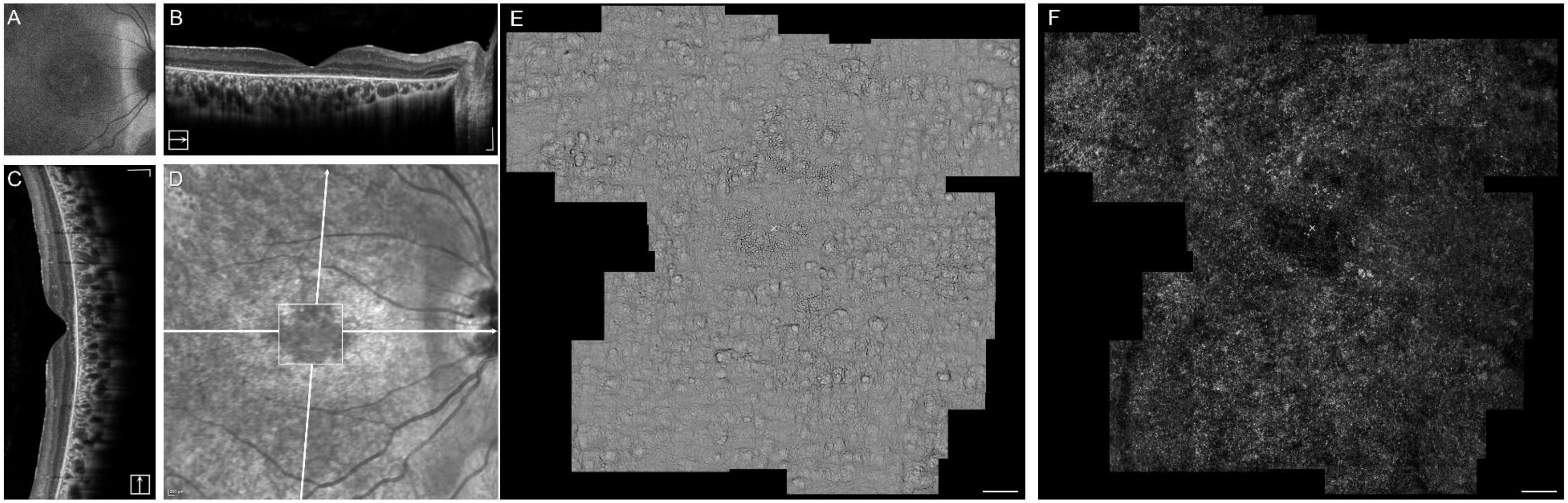

Supplementary Figure 3: Multimodal imaging of the right eye of patient MM_0594 with *RDH12*-EORSD at their first visit. BAF image shown in (**A**). The horizontal and vertical line scans on the NIR reflectance image in (**D**) reflect the locations of horizontal and vertical OCT B-scans (**B** and **C**, respectively), while the white rectangle represents the area where the AOSLO quadrant-detection image (**E**) and confocal image (**F**) were taken. The intersection of the two line scans marks the fovea; X in images (**E**) and (**F**). Scale bars for (**B**), (**C**) and (**D**) = 200 μm. Scale bars for (**E**) and (**F**) = 100 μm.

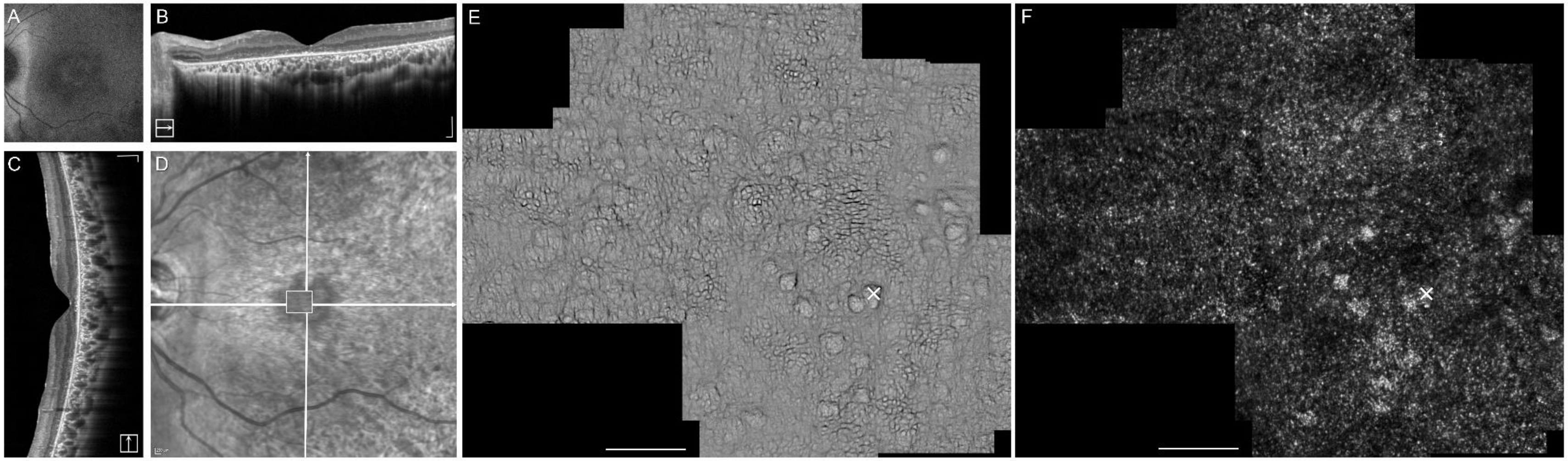

Supplementary Figure 4: Multimodal imaging of the left eye of patient MM_0594 with *RDH12*-EORSD at their first visit. BAF image shown in (**A**). The horizontal and vertical line scans on the NIR reflectance image in (**D**) reflect the locations of horizontal and vertical OCT B-scans (**B** and **C**, respectively), while the white rectangle represents the area where the AOSLO quadrant-detection image (**E**) and confocal image (**F**) were taken. The intersection of the two line scans marks the fovea; X in images (**E**) and (**F**). Scale bars for (**B**), (**C**) and (**D**) = 200 μm. Scale bars for (**E**) and (**F**) = 100 μm.

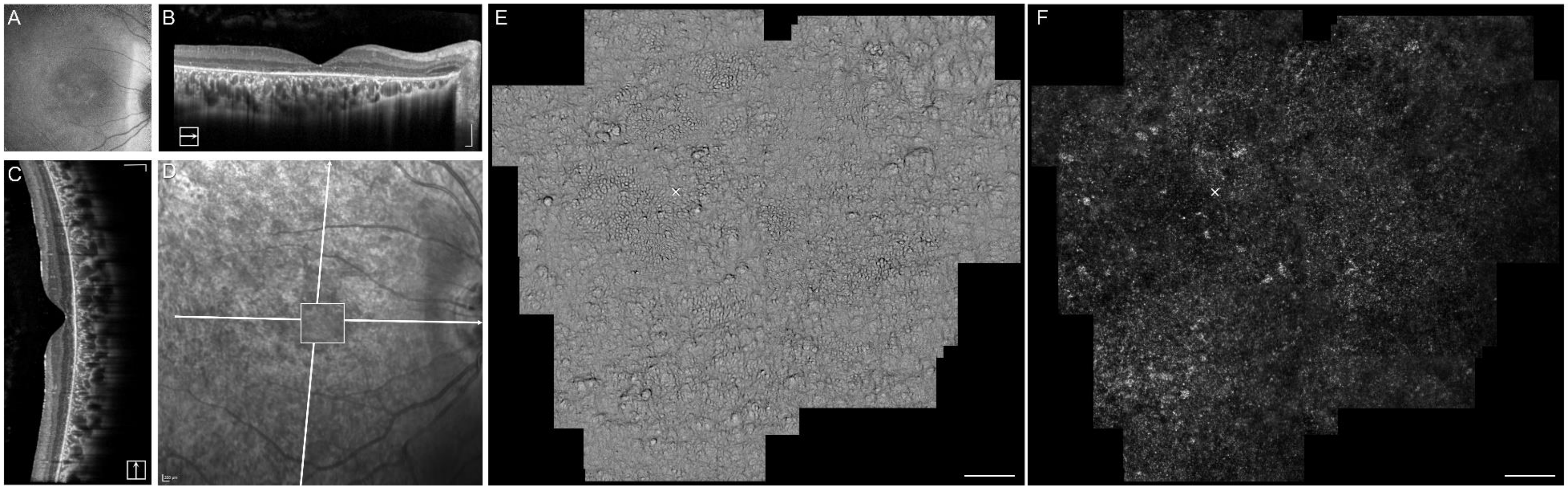

Supplementary Figure 5: Multimodal imaging of the right eye of patient MM_0594 with *RDH12*-EORSD at their second visit (6 months after their first visit). BAF image shown in (**A**). The horizontal and vertical line scans on the NIR reflectance image in (**D**) reflect the locations of horizontal and vertical OCT B-scans (**B** and **C**, respectively), while the white rectangle represents the area where the AOSLO quadrant-detection image (**E**) and confocal image (**F**) were taken. The intersection of the two line scans marks the fovea; X in images (**E**) and (**F**). Scale bars for (**B**), (**C**) and (**D**) = 200 µm. Scale bars for (**E**) and (**F**) = 100 µm.

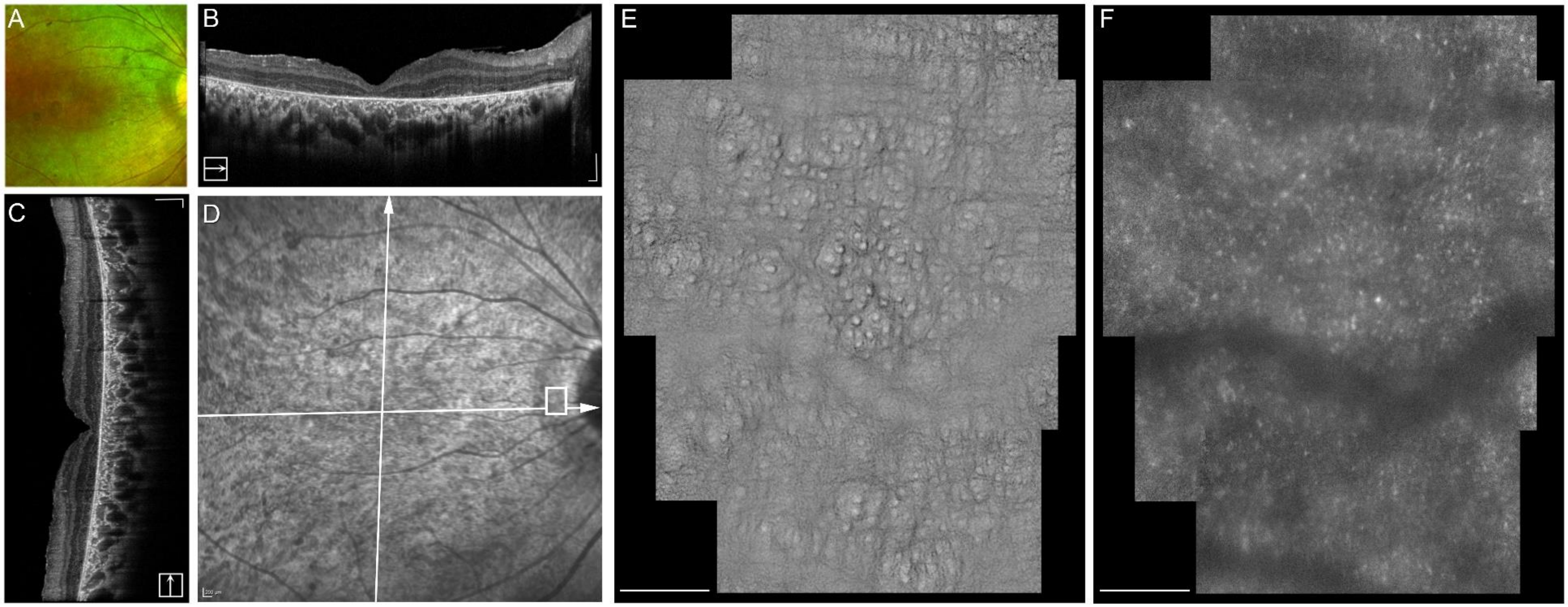

<u>Supplementary Figure 6:</u> Multimodal imaging of the right eye of patient MM_0600 with *RDH12*-EORSD. OPTOS image shown in (**A**) is cropped to reflect the area of the NIR reflectance image in (**D**). The crosshair lines in (**D**) reflect the locations of horizontal and vertical OCT B-scans (**B** and **C**, respectively), while the white rectangle represents the area where the AOSLO quadrant-detection image (**E**) and confocal image (**F**) were taken. Scale bars for (**B**), (**C**) and (**D**) = 200 μm. Scale bars for (**E**) and (**F**) = 100 μm.

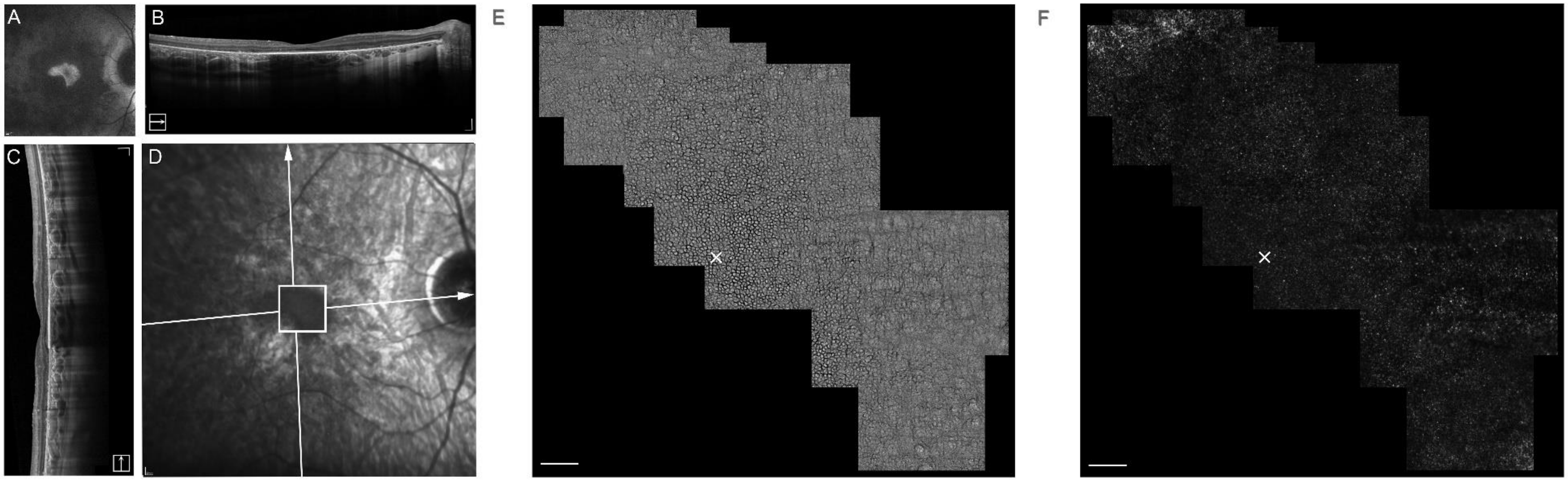

Supplementary Figure 7: Multimodal imaging of the right eye of patient MM_0608 with *RDH12*-EORSD. BAF image shown in (**A**). The horizontal and vertical line scans on the NIR reflectance image in (**D**) reflect the locations of horizontal and vertical OCT B-scans (**B** and **C**, respectively), while the white rectangle represents the area where the AOSLO quadrant-detection image (**E**) and confocal image (**F**) were taken. The intersection of the two line scans marks the fovea; X in images (**E**) and (**F**). Scale bars for (**B**), (**C**) and (**D**) = 200 μm. Scale bars for (**E**) and (**F**) = 100 μm.

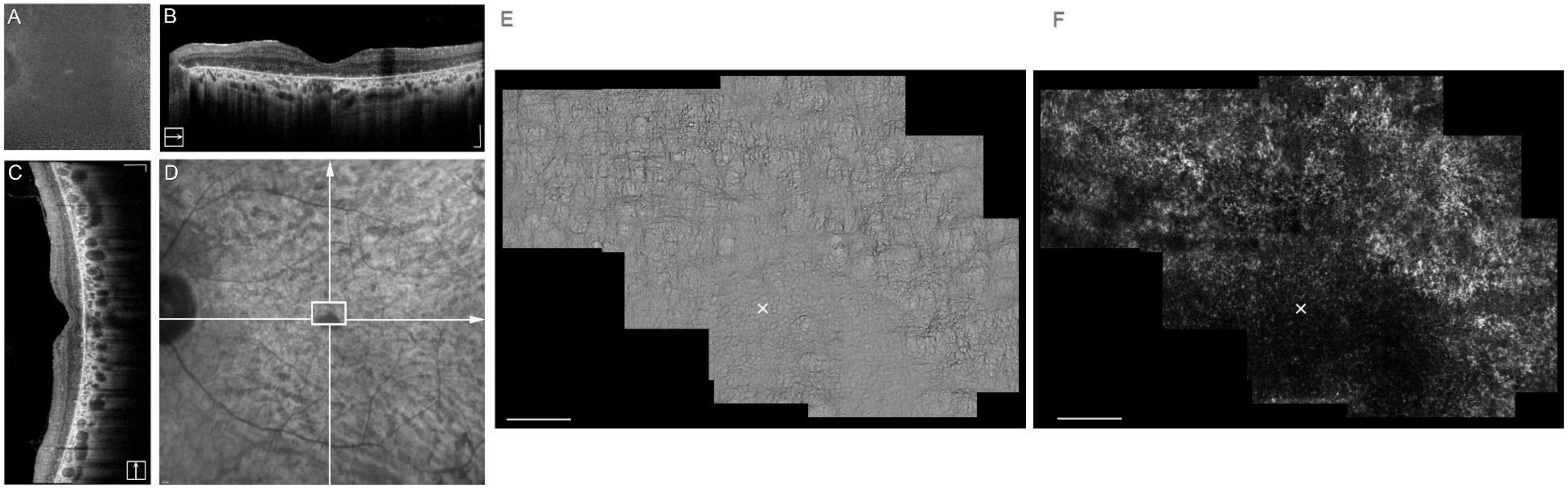

Supplementary Figure 8: Multimodal imaging of the left eye of patient MM_0628 with *RDH12*-EORSD. BAF image shown in (**A**). The horizontal and vertical line scans on the NIR reflectance image in (**D**) reflect the locations of horizontal and vertical OCT B-scans (**B** and **C**, respectively), while the white rectangle represents the area where the AOSLO quadrant-detection image (**E**) and confocal image (**F**) were taken. The intersection of the two line scans marks the fovea; X in images (**E**) and (**F**). Scale bars for (**B**), (**C**) and (**D**) = 200 µm. Scale bars for (**E**) and (**F**) = 100 µm.

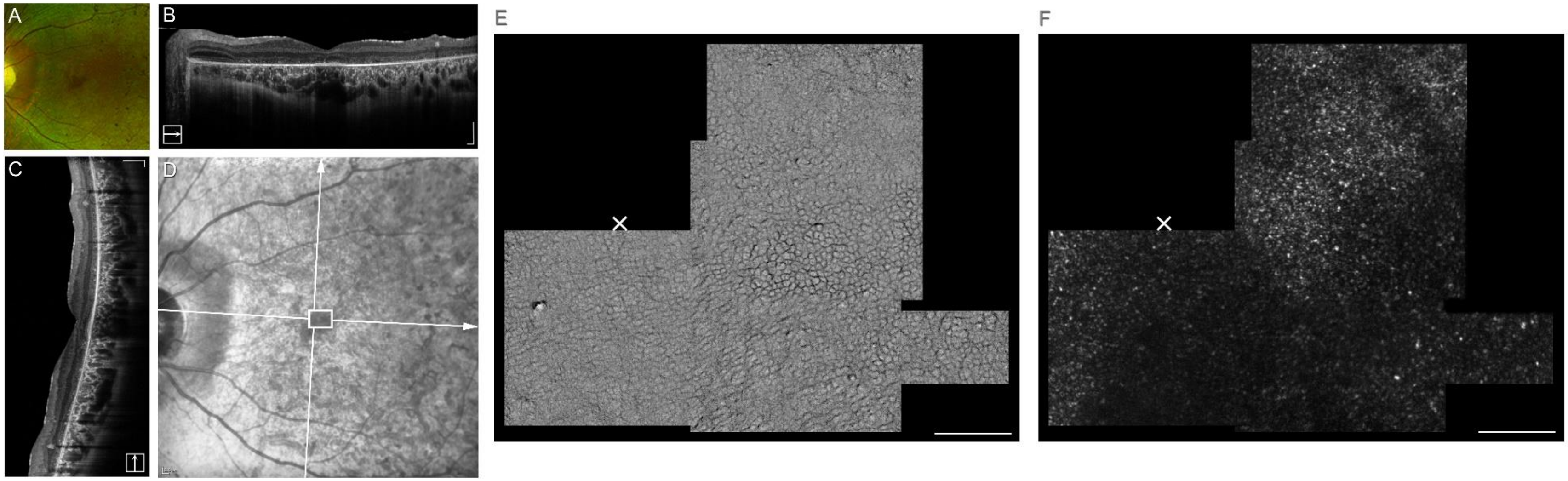

Supplementary Figure 9: Multimodal imaging of the left eye of patient MM_0629 with *RDH12*-EORSD. OPTOS image shown in (**A**) is cropped to reflect the area of the NIR reflectance image in (**D**). The crosshair lines in (**D**) reflect the locations of horizontal and vertical OCT B-scans (**B** and **C**, respectively), while the white rectangle represents the area where the AOSLO quadrant-detection image (**E**) and confocal image (**F**) were taken. The intersection of the two line scans marks the fovea; X in images (**E**) and (**F**). Scale bars for (**B**), (**C**) and (**D**) = 200 μm. Scale bars for (**E**) and (**F**) = 100 μm.

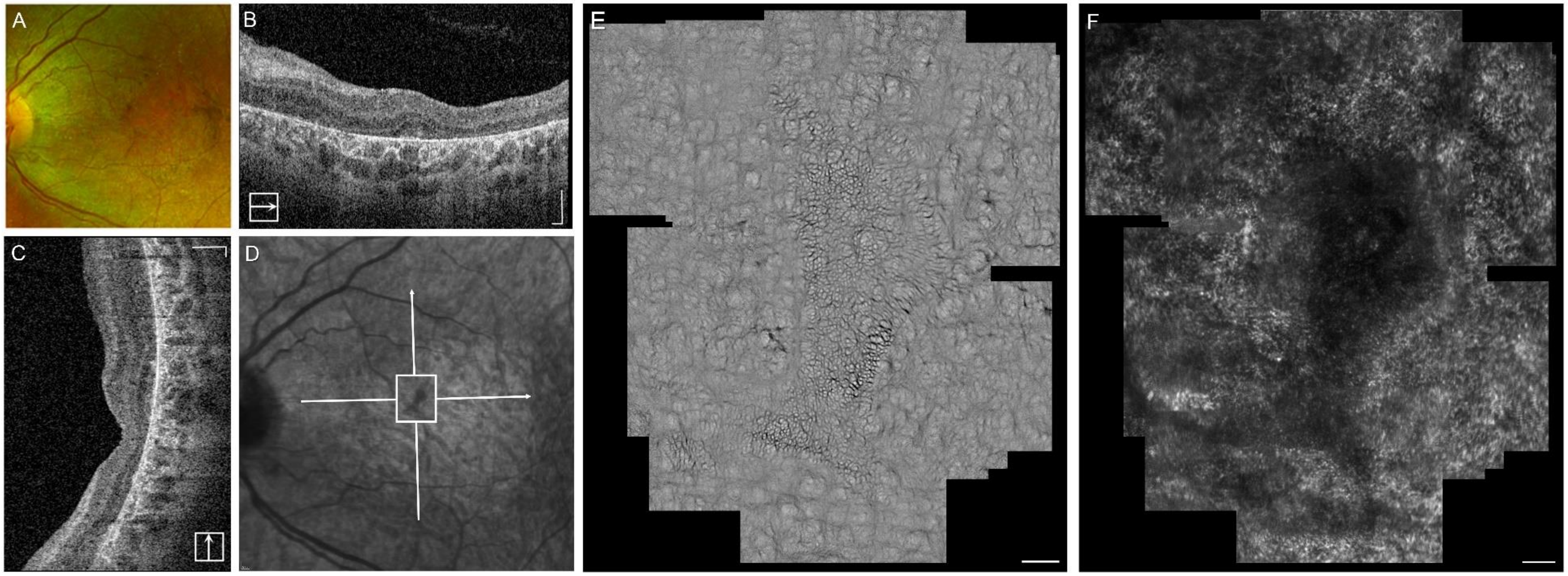

Supplementary Figure 10: Multimodal imaging of the left eye of patient MM_0642 with *RDH12*-EORSD. OPTOS image shown in (**A**) is cropped to reflect the area of the NIR reflectance image in (**D**). The horizontal (**B**) and vertical (**C**) OCT B-scans belong to a different timepoint from the one that AOSLO images were acquired and are not conclusive of the precise foveal centre, therefore there is no X marking the fovea. The white rectangle in (**D**) represents the area where the AOSLO quadrant-detection image (**E**) and confocal image (**F**) were taken. Scale bar for (**D**) = 200 μm. Scale bars for (**E**) and (**F**) = 100 μm.
